# A Hybrid Diagnostic Framework with Compensation Algorithms for Inherent Rotor Faults Using Rotor Experiments

**DOI:** 10.3390/s26082565

**Published:** 2026-04-21

**Authors:** Shyh-Chin Huang, Thanh-Trung Pham, Trong-Du Nguyen, Yu-Jen Chiu

**Affiliations:** 1 Department of Mechanical Engineering, Ming Chi University of Technology, New Taipei City 24301, Taiwan; m13118032@mail2.mcut.edu.tw (T.-T.P.); yjchiu@mail.mcut.edu.tw (Y.-J.C.); 2 Intelligent Technical Diagnostic Lab, School of Mechanical Engineering, Hanoi University of Science and Technology, Hanoi 100000, Vietnam; du.nguyentrong@hust.edu.vn

**Keywords:** simultaneous imbalance and shaft-bow, inherent faults, hybrid approach, self-compensations, rotor faults diagnosis

## Abstract

In practical engineering applications, rotor–bearing systems inevitably exhibit inherent or residual faults such as imbalance and shaft-bow, originating from manufacturing tolerances, thermal deformation, or operational loading. Accurate monitoring of these faults and their evolution is fundamental to the effectiveness of modern prognostics and health management (PHM) frameworks. However, if such inherent faults are not identified at an early stage, substantial deviations in fault diagnosis may occur, thereby compromising the accuracy of subsequent prognostic assessments and maintenance strategies. This study presents a hybrid diagnostic methodology that integrates a physics-based model with neural network techniques to enhance rotor fault diagnosis. A Jeffcott rotor subjected to simultaneous disk imbalance and shaft-bow is used to demonstrate the methodology, and the results proves its superior capability for simultaneous fault identification. Nonetheless, discrepancies between model predictions and experimental results are observed, attributed to the presence of inherent faults within the rotor system. To address this issue, algorithms for inherent fault identification and compensation, supported by experimental verification, are developed. Following compensation, the accuracy in simultaneously diagnosing and estimating the parameters of imbalance and shaft-bow is significantly improved. The proposed methodology is designed for seamless integration into real-time monitoring systems of industrial rotating machinery.

## 1. Introduction

In today’s industries, rotating machines are the core of many important areas such as manufacturing, power generation, petrochemical plants, and transportation [1,2]. For these systems, smooth and reliable operation is crucial to keep production running, protect worker safety, and reduce costs in the long term. Over time, maintenance strategies have developed from reactive maintenance, where faults are fixed only after breakdowns happen, to preventive maintenance, which follows fixed schedules, and more recently to predictive maintenance (PdM). Predictive maintenance uses advanced condition monitoring (CM) methods to track key parameters like vibration, temperature, and lubricant condition while the machine is working [3]. By doing so, it helps detect early warning signs of problems before serious damage occurs. This proactive approach reduces unexpected downtime, increases the service life of equipment, saves maintenance resources, and improves safety, making it an essential practice in Industry 5.0 [4].

Among the various components in rotating machinery, the rotor shaft-disk plays a central role in systems such as turbines, compressors, electric generators, and machine tools, where it is often exposed to complex dynamic loads and harsh operating conditions [5]. Faults such as disk imbalance, shaft-bow, shaft cracks, and bearing defects can alter the rotor’s dynamic behavior, causing vibration anomalies that propagate to other subsystems and, in severe cases, lead to catastrophic breakdowns with considerable economic and safety consequences [6].

Analytical models have long been utilized to investigate rotor dynamics and fault propagation, serving as a theoretical basis for developing diagnostic algorithms and compensation strategies. To detect and assess such faults, a range of diagnostic methods has been established, encompassing temperature monitoring for overheating and lubrication degradation, acoustic emission and ultrasonic testing for detecting crack initiation and wear, lubricant analysis for identifying contaminants and wear particles, electromagnetic techniques for detecting corrosion and cracks, and torque monitoring for evaluating overloads and efficiency losses [7]. While each technique offers specific advantages, none can comprehensively identify all fault types. This limitation has encouraged the integration of multiple sensing modalities in condition monitoring to capture a more complete picture of machine health. However, real-world signals are frequently contaminated by noise and influenced by varying operating conditions, which complicates both feature extraction and interpretation.

Vibration analysis is now the most widely used method to check the condition of rotating machines because it can detect even very slight changes in structure or operation [8]. Since different faults generate distinct vibration characteristics, vibration-based diagnostics (VBD) is effective for identifying many rotor problems [9]. In general, VBD methods are divided into three groups [10]: model-based methods, which use physical and mathematical models; data-driven methods, which use measured data and algorithms; and hybrid methods, which combine both. These approaches help detect problems early, improve maintenance planning, and ensure reliable operation.

Imbalance is one of the faults that VBD can detect, and it has received the most attention because a perfectly balanced machine does not exist. Small errors in geometry, assembly, or material quality always create imbalance, which leads to unwanted vibrations and reduced performance. Many studies have focused on imbalance, including its interaction with misalignment [11], diagnostic methods [12], modeling approaches, and experiments. A related fault is shaft-bow, which may occur temporarily or permanently due to gravity, uneven heating or cooling, or deformation caused by long-term imbalance. Earlier studies show that imbalance and shaft bowing strongly influence each other: Nicholas et al. [13] studied the effect of residual bow on rotor response, Shiau and Lee [14] analyzed rotors with combined disk skew, imbalance, and bow, and Song et al. [15] simulated how residual bow affects longitudinal response. These results confirm that the two faults should be studied together in diagnostics and modeling. Identifying the inherent residual imbalance or shaft-bow individually is relatively straightforward. However, quantitatively distinguishing the simultaneous presence of both faults presents significant challenges due to the non-uniqueness of possible combinations. This is because both faults excite vibrations at the same frequency, making it difficult to separate their contributions.

Based on these fault mechanisms, many data-driven methods have been developed. They are usually divided into signal processing and machine learning. Signal processing methods focus on extracting useful features from vibration signals. For example, Nguyen et al. [16] used filtering and synchrosqueezing to extract fault features; He et al. [17] proposed an adaptive Variational Mode Decomposition (VMD) optimized by metaheuristic algorithms; Chegini et al. [18] applied wavelet-based denoising to improve early detection; Wang et al. [19] introduced a sparsity-guided EWT to preserve resonance bands; and Li et al. [20] combined improved VMD with sparse coding to reduce noise and extract impulses. These methods show the importance of advanced signal processing in reliable fault detection. Machine learning methods, often supported by artificial intelligence (AI), can automatically learn fault patterns from large datasets without detailed physical models. Altaf et al. [21] achieved over 99% accuracy with statistical and spectral features; Sohaib et al. [22] combined hybrid features with deep neural networks to recognize different levels of fault severity; and Deng et al. [23] used wavelet features with Support Vector Machine (SVM) to achieve high accuracy even on small datasets. Similarly, Liang et al. [24] applied vibration-based features with PCA and a GWO–ELM model to predict the remaining useful life of packing sets in high-pressure plunger pumps, further demonstrating the effectiveness of data-driven methods in practical prognostics. These approaches are powerful but depend on large and diverse training data, which are often difficult to collect in real industrial environments [25].

To deal with this concern, many researchers turn to model-based methods. Unlike data-driven approaches that depend heavily on large datasets, these methods build physical or mathematical models to simulate how machines behave under different fault conditions. This not only reduces the need for extensive training data but also provides deeper physical insight, making the diagnostic results more interpretable and easier to validate. For example, Lin et al. [26] proposed a two-step model for rotor imbalance, which was successfully validated on long-term industrial data, and Bera et al. [27] developed an adaptive model that updates its parameters through optimization and forecasting, allowing for accurate fault tracking as conditions change. Earlier works also demonstrate the strength of this approach: Pennacchi and Vania [28] modeled thermal bow in turbines, and Shrivastava et al. [29] combined Kalman filtering with recursive least squares for unbalance estimation. Similar approaches have also been reported, such as physics-based modeling of rotating shafts for anomaly detection [30], and hybrid gray-box schemes that combine analytical modeling with limited measurement data [31]. Together, these studies confirm that model-based methods can achieve accurate diagnosis while offering valuable physical understanding of rotor dynamics. However, despite their proven effectiveness, these methods require precise determination of system parameters and often face challenges in representing nonlinear characteristics and parameter uncertainties. In addition, constructing and validating such models is usually time-consuming and demands specialized expertise, which limits their practical application in complex industrial environments.

In this context, hybrid approaches have gained increasing attention for rotor fault diagnosis because they combine the interpretability of physics-based models with the adaptability of data-driven methods. It is worth noting that the term hybrid is sometimes used ambiguously in the literature. In some cases, it merely refers to the combination of multiple feature extraction techniques (e.g., time, frequency, and wavelet-based indicators) with machine learning classifiers. While such strategies can enrich the feature space, they remain purely data-driven as they do not integrate physical modeling. In contrast, the hybrid approaches considered in this study explicitly couple physics-based models with data-driven or AI techniques, offering diagnostic frameworks that are both robust and flexible. Previous studies have explored this paradigm from different angles: Leturiondo et al. [32] employed semi-supervised hybrid learning to detect faults with limited labeled data, Sadoughi et al. [33] introduced a physics-informed CNN to incorporate domain knowledge into model training, and Habbouche et al. [34] developed a digital twin framework for gearbox fault diagnosis. Most relevant to this work, Huang et al. [35] presented a hybrid framework that integrates a Jeffcott rotor model with artificial neural networks. By generating simulated datasets from the rotor model, they trained a feed-forward network capable of diagnosing simultaneous imbalance and shaft-bow. Their study demonstrates the effectiveness of combining physics-based simulation with machine learning to improve diagnostic accuracy in complex fault scenarios. Their results show that high accuracy is achieved in fault diagnosis using simulated data. However, significant errors are observed when the method is validated with an experimental rotor rig. One possible cause could be the presence of inherent imbalance and shaft-bow in the rotor rig, which contribute to the overall response and impact the diagnostic results. The present research is inspired by [35] and aims to develop self-compensation algorithms to identify inherent faults in real rotor systems before any diagnostic analysis can be performed.

A significant and often overlooked challenge in both model-based and data-driven fault diagnosis is the presence of inherent faults, which already exist within a mechanical system before any monitoring or testing begins. These faults may arise from manufacturing inaccuracies, material defects, assembly misalignments, long-term wear, or subtle deformations accumulated during the machine’s service life [36,37]. Unlike faults that develop during operation, inherent faults are embedded in the system’s baseline state and often produce weak or ambiguous vibration signatures. They can subtly alter the dynamic behavior of the machine, shift operating parameters, and mask or distort the signs of other emerging faults [38]. Because these faults are unknown to operators, absent from historical datasets, and excluded from the assumptions of physical models, they introduce an additional layer of uncertainty, making conventional diagnostic techniques less effective [39,40]. In practical terms, this means that even if a machine appears “healthy,” its baseline contains hidden errors that can propagate and interfere with accurate detection of new faults.

To address this challenge, self-compensation algorithms have been developed as a targeted approach for handling inherent faults. These algorithms are designed to identify and quantify the hidden fault components by comparing the measured system responses with the predictions of physical or hybrid models. The deviations between predicted and actual measurements are interpreted as contributions from inherent faults, which can then be isolated and quantified. Once determined, these inherent fault values are integrated into the diagnostic process as corrective factors, effectively “compensating” for the baseline errors that would otherwise distort fault detection. The self-compensation process is dynamic and iterative, continuously updating fault estimates in real time as new measurement data becomes available. By doing so, it filters out the influence of unknown baseline errors, enhancing both the precision of active fault detection and the overall reliability of the monitoring system. The connection between inherent faults and self-compensation is critical: inherent faults represent the hidden baseline errors, while self-compensation provides the mechanism to detect and correct for these errors, allowing hybrid diagnostic systems to produce more accurate and robust assessments of machine health. This approach is particularly valuable in industrial applications where machines often operate under varying conditions and where baseline deviations could otherwise mislead predictive maintenance decisions. By integrating self-compensation algorithms with hybrid models-combining physics-based simulations with data-driven learning-engineers can simultaneously account for known operational faults and hidden inherent errors, ensuring that maintenance actions are based on a clearer and more complete understanding of the machine’s true condition.

In this study, we propose a hybrid method and demonstrate through a Jeffcott rotor with a feed-forward neural network to diagnose and estimate the parameters of simultaneous imbalance and shaft-bow. The physical model generates simulated datasets for training, while the trained network processes experimental vibration data. In addition, a self-compensation algorithm is developed to identify inherent faults present within the experimental rig. The values of these inherent faults are then used to compensate the diagnosis process, improving accuracy and robustness. This integrated approach ensures precise fault identification, enhances reliability, and extends the service life of critical rotating machinery.

This study makes the following main contributions:

- A hybrid physics–AI framework is proposed for the simultaneous estimation of rotor imbalance and shaft-bow parameters.
- A self-compensation algorithm is introduced to identify inherent faults in real rotor systems and to improve the diagnostic reliability.
- Two compensation schemes are formulated and experimentally compared.
- Experiments on an RK4 Bently Nevada rotor rig demonstrate improved diagnostic accuracy, supporting applicability to practical condition monitoring of rotating machinery.

The remainder of this paper is organized as follows: Section 2 describes the hybrid approach and the research motivation; Section 3 presents the self-compensation algorithm; Section 4 provides the experimental validation and discussion; and Section 5 concludes the paper.

## 2. A Hybrid Approach for Rotor Imbalance and Shaft-Bow Diagnosis

This section is entirely based on the knowledge presented in the work of Huang et al. [35], who developed a hybrid framework combining physical modeling of a rotor system with machine learning techniques for fault diagnosis. Their study provided both the theoretical foundation and methodological guidance that are adopted in the present research.

**Figure 1:**
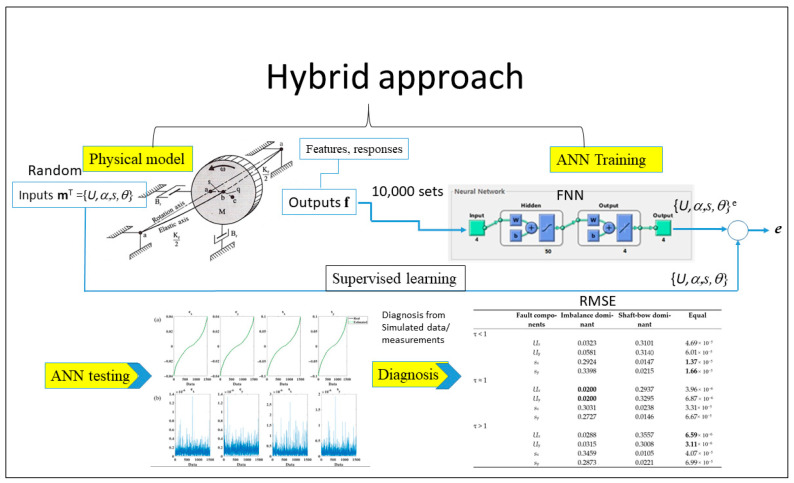
Flowchart of hybrid framework for imbalance and shaft-bow diagnoses.

According to the hybrid methodology outlined in [35], the process begins with the construction of a simplified rotor model that incorporates the effects of imbalance and shaft-bow. The vibration responses obtained from this model are then transformed into fault features. By systematically varying the fault parameters, synthetic fault feature datasets can be generated, which are subsequently used to train a machine learning model. Once trained, this model is applied to experimental vibration signals, where it estimates the underlying fault parameters. In this way, the hybrid approach integrates the interpretability of physical modeling with the adaptability of data-driven learning. The flowchart of this hybrid approach is briefly illustrated in Figure 1.

### 2.1. Physical Model of a Jeffcott Rotor

**Figure 2:**
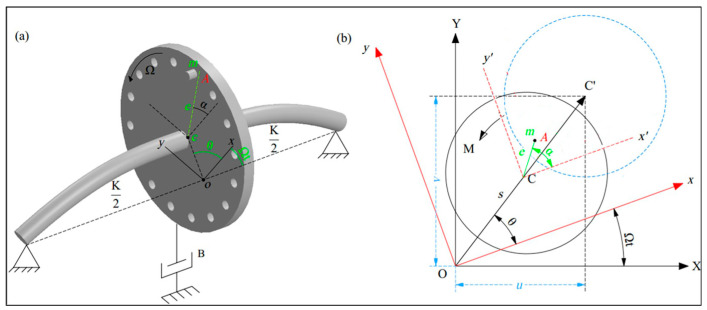
Schematic of Jeffcott rotor: (**a**) with simultaneous imbalance and shaft-bow faults; (**b**) the disk geometric relations.

The physical modeling is based on the Jeffcott rotor, as shown in Figure 2, which consists of a rigid disk of mass M mounted at the midpoint of a massless flexible shaft supported by bearings. The serial combination of shaft and bearing provides a total stiffness of K and damping constant of B. Two types of faults are considered: imbalance, introduced by an eccentric mass m at a distance e from the disk center with orientation angle α, and shaft-bow, represented by a static deflection s oriented at an angle θ. Both angles are measured with respect to the key phasor (KP). The solid circle represents the initial position of rotating disk. The dashed circle denotes the displaced position. X−Y, x−y, and $x^{\prime }-y^{\prime }$ axes correspond to the fixed, rotating, and disk-fixed coordinate systems, respectively. Different colors are used solely for clarity.

Denoting the lateral displacements of the disk center by u(t) and v(t) in the horizontal and vertical directions, respectively, the equations of motion can be expressed as:

(1) $$\left(M+m\right)\ddot{u}+B_{x}\dot{u}+K_{x}u=K_{x}s\cos\left(\Omega t+\theta \right)+me\Omega^{2}\cos\left(\Omega t+\alpha \right)$$

(2) $$\left(M+m\right)\ddot{v}+B_{y}\dot{v}+K_{y}v=K_{y}s\sin\left(\Omega t+\theta \right)+me\Omega^{2}\sin\left(\Omega t+\alpha \right)$$

The right-hand sides of Equations (1) and (2) represent excitation due to shaft-bow and imbalance. Both excitations occur at the same frequency, Ω, which makes the diagnosis non-unique. Therefore, a hybrid approach that integrates theoretical derivation and an AI-based model is developed to resolve this uncertainty. The responses in the X and Y directions can be solved from (1) and (2) as follows:

(3) $$u\left(t\right)=A_{x}\left[\frac{U\tau_{x}^{2}}{M_{T}}\cos\left(\Omega t+\alpha -\lambda_{x}\right)+s\cos\left(\Omega t+\theta -\lambda_{x}\right)\right]$$

(4) $$v\left(t\right)=A_{y}\left[\frac{U\tau_{y}^{2}}{M_{T}}\sin\left(\Omega t+\alpha -\lambda_{y}\right)+s\sin\left(\Omega t+\theta -\lambda_{y}\right)\right]$$

where $A_{x}$ is the so-called amplification factor as:

(5) $$A_{x}=\frac{1}{\sqrt{{\left(1-\tau_{x}^{2}\right)}^{2}+{\left(2\tau_{x}\lambda_{x}\right)}^{2}}}$$

(6) $$\tau_{x}=\frac{\Omega }{\omega_{nx}}$$

(7) $$\lambda_{x}=\tan^{-1}\left(\frac{2\zeta_{x}\tau_{x}}{1-\tau_{x}^{2}}\right)$$

where τ is the speed ratio or frequency ratio and λ is the response phase lag. Given that m, the imbalance mass, is negligible compared to M(m<<M) in most situation, the total mass $M_{T}$ can be approximated as M without significant loss of accuracy.

The responses of Equations (3) and (4) can be further rearranged in terms of the $\cos\left(\Omega t\right)$ and $\sin\left(\Omega t\right)$ components as follows:

(8) $$u\left(t\right)=f_{1}\cos\left(\Omega t\right)+f_{2}\sin\left(\Omega t\right)$$

(9) $$v\left(t\right)=f_{3}\cos\left(\Omega t\right)+f_{4}\sin\left(\Omega t\right)$$

where $f_{1},f_{2},f_{3}$ and $f_{4}$ are the four features as functions of the four fault variables U,α,s and θ, respectively, defined as:

(10) $$f_{1}=A_{x}\left[\frac{U\tau_{x}^{2}}{M}\cos\left(\alpha -\lambda_{x}\right)+s\cos\left(\theta -\lambda_{x}\right)\right]$$

(11) $$f_{2}=-A_{x}\left[\frac{U\tau_{x}^{2}}{M}\sin\left(\alpha -\lambda_{x}\right)+s\sin\left(\theta -\lambda_{x}\right)\right]$$

(12) $$f_{3}=A_{y}\left[\frac{U\tau_{y}^{2}}{M}\sin\left(\alpha -\lambda_{y}\right)+s\sin\left(\theta -\lambda_{y}\right)\right]$$

(13) $$f_{4}=A_{y}\left[\frac{U\tau_{y}^{2}}{M}\cos\left(\alpha -\lambda_{y}\right)+s\cos\left(\theta -\lambda_{y}\right)\right]$$

or in terms of the feature vector as:

(14) $$f_{4\times 1}={\left\{f_{1},f_{2},f_{3},f_{4}\right\}}^{T}$$

The fault variables associated with imbalance and shaft-bow can be written as a fault vector:

(15) $$m_{4\times 1}={\left\{U,\alpha ,s,\theta \right\}}^{T}$$

Based on Equations (3) and (4), at each time *t* and for a given rotational speed Ω, the system parameters τ and λ can be readily calculated. Together with Equations (10)–(13), this shows that for each fault vector $m_{4\times 1}$, the corresponding feature vector $f_{4\times 1}$ can be obtained from the physical model. Using this property, new datasets can be generated through the physical model.

### 2.2. AI Model Set up and Simulation Tested Result

**Figure 3:**
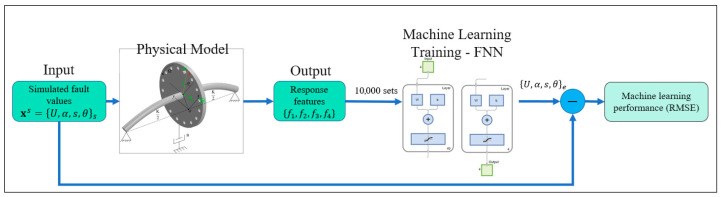
The process of artificial intelligence model construction.

The construction and training of an Artificial Intelligence (AI) model is illustrated in Figure 3. To develop a sufficiently rich dataset for training, a total dataset of 10,000 fault cases was synthetically generated through randomized sampling of fault parameters. The imbalance magnitude was varied within the range of 0 to 1000 g·mm, while the shaft-bow amplitude was selected from 0 to 50 mm. A shaft-bow of 50 mm is not physically realistic for practical rotating machinery. However, the influence of shaft-bow amplitude on the vibration response is essentially linear, as indicated in Equations (10)–(13). In our preliminary tests, both wider and narrower ranges of shaft-bow amplitudes were examined, and the results showed no significant effect on the accuracy of the FNN model. For this reason, the shaft-bow range was intentionally extended to a relatively larger range in order to increase the diversity of the training dataset and improve the robustness of the learning process. Additionally, both the imbalance angle and shaft-bow orientation were uniformly distributed between 0° and 360°. These randomly generated fault parameters were subsequently substituted into the governing mathematical formulations of the rotor system to compute the corresponding feature sets. The resulting dataset that includes all relevant dynamic features was then used as the input for AI model training.

A feed-forward neural network (FNN) architecture was adopted for the regression task. The network consists of a single hidden layer with 40 neurons, and its parameters were optimized using the Bayesian Regularization backpropagation algorithm. This training algorithm was selected due to its ability to reduce overfitting and improve generalization performance, especially when dealing with noisy or highly nonlinear relationships. The maximum number of training epochs was set to 1000. During the training process, the dataset was partitioned with 70% allocated for training, 15% for testing, and another 15% for validation to ensure independent performance evaluation. The selected network architecture follows the configuration proposed in our previous work [35], in which different numbers of hidden neurons were systematically evaluated. The results of that study showed that a single hidden layer with 40 neurons provides the best compromise between prediction accuracy and computational efficiency for the simple Jeffcott rotor. Nevertheless, more complex neural network architectures may be required when dealing with more complicated rotor systems.

To quantitatively assess the predictive capability of the developed AI model, the Root Mean Squared Error (RMSE) was employed as the performance indicator. RMSE provides a direct measure of the average prediction error between the estimated and actual fault values, where a lower RMSE corresponds to higher model accuracy and improved diagnostic performance. The RMSE is mathematically expressed as:

(16) $$RMSE=\sqrt{\frac{1}{n}\sum_{i=1}^{n}{\left(x_{i}-\hat{x_{i}}\right)}^{2}}$$

where $x_{i}$ denotes the true value of the fault parameter, $\hat{x_{i}}$ is the corresponding predicted value from the AI model, and n represents the total number of samples.

To facilitate the quantitative evaluation of diagnostic errors and to enable a clear representation in terms of percentage values, the fault parameters are expressed in terms of their fault components rather than their amplitude-phase form. Specifically, instead of using the fault values (U,α,s,θ), the corresponding fault components ($U_{x},U_{y},S_{x},S_{y}$) are adopted in this study. This transformation avoids ambiguity caused by the periodic nature of phase angles and ensures a consistent error definition when comparing diagnosed and reference values. The fault components are obtained from the fault values according to the following relationships:

(17) $$U_{x}=U\cos(\alpha )$$

(18) $$U_{y}=U\sin(\alpha )$$

(19) $$S_{x}=s\cos(\theta )$$

(20) $$S_{x}=s\sin(\theta )$$

**Figure 4:**
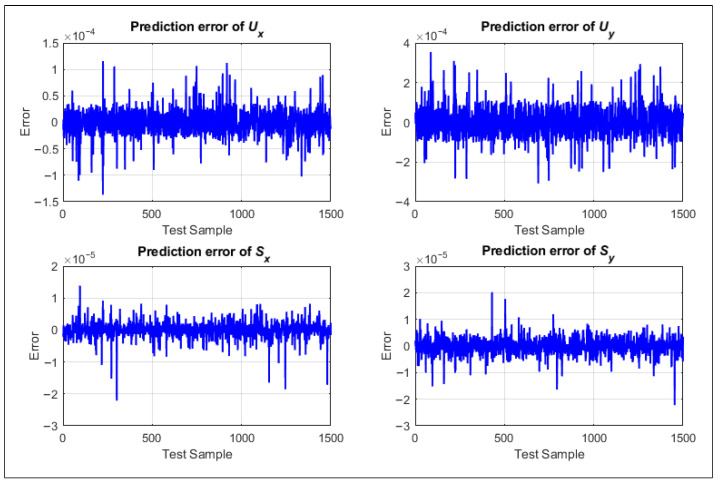
Distributed prediction errors of four fault components over the test samples.

The prediction error histories of the four fault components $U_{x},U_{y},S_{x}$, and $S_{y}$ show that the deviations between the predicted and reference values remain tightly clustered around zero over the entire test set, without any visible drift or systematic trend. The error amplitudes for the imbalance-related components $U_{x}$ and $U_{y}$ are on the order of $10^{-4}$, whereas those for the shaft-bow components $S_{x}$ and $S_{y}$ are even smaller, on the order of $10^{-5}$, indicating that the trained AI model exhibits good generalization capability and stable behavior when evaluated on a large number of unseen samples (as shown in Figure 4). The dense and nearly symmetric distribution of error points around the zero line further suggests that the residual deviations are predominantly random and noise-related rather than arising from a structural bias of the model.

These qualitative observations are quantitatively supported by the average RMSE values reported in Table 1. Such small RMSE magnitudes are significantly lower than the characteristic amplitudes of the vibration features used for training, confirming that the AI model can reconstruct the fault-related components with very high accuracy. Moreover, the fact that the shaft-bow components achieve RMSE values nearly one order of magnitude smaller than those of the imbalance components indicates that the network is particularly sensitive to subtle variations in bow-related features.

Taken together, the small prediction errors and low RMSE values demonstrate that the trained AI model provides a sufficiently precise mapping from feature values to fault parameters and can therefore be reliably employed in the subsequent stages of the real experiment data diagnosis.

### 2.3. Validation of Experiment Data

#### 2.3.1. Experiment Setup

**Figure 5:**
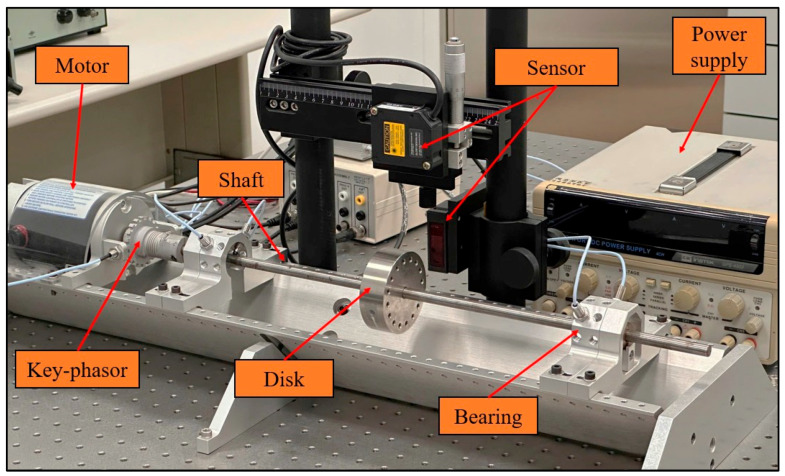
Experimental setup of the Jeffcott rotor system.

The rotor fault experiments in this study were conducted using the RK4 Bently Nevada experimental rig, as illustrated in Figure 5. The rig was specifically designed to emulate the fundamental dynamic characteristics of rotor–bearing systems under controlled laboratory conditions, providing a versatile platform for investigating imbalance and shaft-bow faults. The overall dimensions of the experimental system are 310 × 135 × 80 mm. The setup consists of an electric motor directly coupled to a single-disk rotor, which is driven in a counterclockwise direction when viewed from the motor side. The rotor shaft, with a length of 240 mm and a mass of 350.62 g, has a diameter of 10 mm and was manufactured with sufficient rigidity to sustain high rotational speeds while remaining sensitive to fault-induced vibrations. The shaft is supported by two identical journal bearings whose stiffness values are not precisely known, thereby introducing a realistic level of uncertainty into the experimental system. A detailed procedure for handling this bearing stiffness uncertainty can be found in the work of Huang et al. [35], where a comprehensive parameter identification approach was presented based on the measured first critical speed.

For accurate vibration monitoring, high-resolution Memstec Glory Laser CD3S-30 and CD3S-50 displacement sensors (MEMSTEC Technology Corp., New Taipei City, Taiwan) were precisely positioned along the orthogonal X- and Y-axes of the disk, separated by an angle of 90°. This configuration ensured comprehensive measurement of the rotor’s lateral motion and dynamic response in two perpendicular directions. Following installation, the sensors were carefully integrated into a stable power supply and data acquisition system, guaranteeing reliable, uninterrupted operation and minimizing the possibility of measurement errors. The entire setup provided a robust foundation for systematically investigating the dynamic behavior of the rotor system under varying fault scenarios.

**Figure 6:**
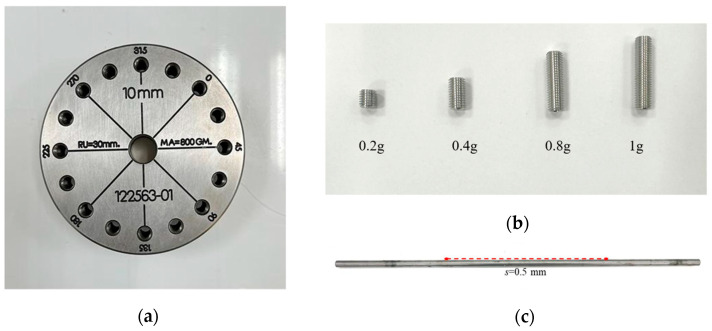
(**a**) The rotating disk consists of 16 holes to attach the imbalance masses. (**b**) The four imbalance masses are 0.2 g, 0.4 g, 0.8 g, and 1.0 g. (**c**) The amount of shaft-bow is 0.5 mm.

Figure 6 illustrates the experimental configuration used to introduce controlled imbalance and shaft-bow into the rotor system. As shown in Figure 6a, a rigid disk, measuring 75 mm in diameter and weighing approximately 800 g, was firmly mounted onto the shaft using radial screws. To enable the controlled introduction of artificial imbalance, the disk was manufactured with 16 symmetrically distributed tapped holes, located on both sides of the disk face at a radial distance of e = 30 mm. These holes allowed the attachment of additional mass in a flexible and repeatable manner, thereby simulating different levels of unbalance severity. Four cylindrical imbalance masses are employed in the experiments, with weights of 0.2 g, 0.4 g, 0.8 g, and 1.0 g, as displayed in Figure 6b. These correspond to normalized imbalance parameters of U = 6, 12, 24, and 30, respectively, depending on the selected mass and radial placement. To simulate shaft-bow, the rotor shaft was intentionally deformed with a static offset of 0.5 mm, as depicted in Figure 6c. Together, these configurations enable systematic testing of rotor dynamics under varying fault conditions, including different levels of imbalance and bowing.

In the experimental setup, the rotating shaft was operated under steady-state conditions at two constant rotational speeds: a sub-critical speed of 1500 rpm and a trans-critical speed of 2500 rpm. The vibration signals were acquired simultaneously from two sensors, both of which were configured with a sampling frequency of 2 kHz to ensure enough temporal resolution for fault characterization. In this study, four distinct fault cases were systematically investigated to evaluate the performance and robustness of the proposed diagnostic approach. The detailed parameters associated with each fault condition are summarized in Table 2. In this experimental study, four different fault cases were investigated, namely the Same quadrant, In-phase, Anti-phase, and Perpendicular configurations.

For all four test cases, the shaft-bow fault parameters were kept unchanged, with a shaft-bow amount s = 0.5 mm and a shaft-bow angle θ = 45°. This decision was mainly based on practical experimental considerations. In practice, it is difficult to fabricate shaft-bow with high accuracy and good repeatability. In addition, changing the shaft-bow angle requires removing the disk from the shaft and rotating it to a specific angular position relative to the key phasor, which makes the experimental procedure more complicated and introduce some uncertainties. To maintain data consistency throughout the measurement and data collection process, only the imbalance fault parameters were varied in this study. The imbalance level can be easily adjusted by changing the attached mass and installing it into pre-drilled holes on the disk. This method is simpler and more repeatable compared with modifying the shaft-bow fault parameters, and it allows reliable generation of multiple fault cases for evaluating the proposed diagnostic approach.

**Figure 7:**
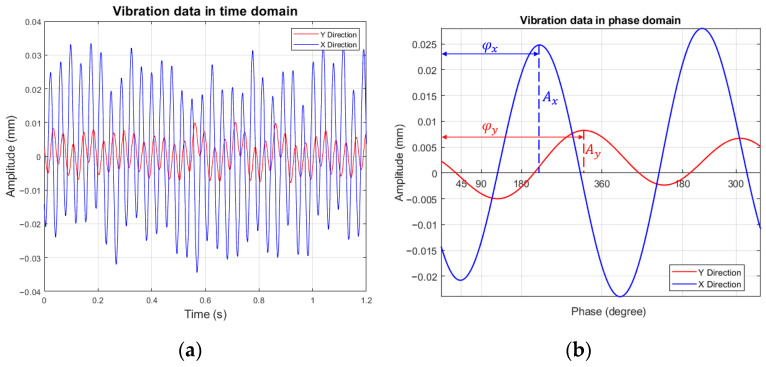
(**a**) Time-based and (**b**) absolute phases of the two laser sensors.

Table 3 summarizes the identified dynamic parameters of the experimental rig in the horizontal (X) and vertical (Y) directions, following the procedure described in Huang et al. [35], including the equivalent mass, $M_{eq}$; damping ratio, ζ; stiffness components, shaft $K_{s}$ and bearing $K_{b}$; and the corresponding natural frequencies, $\omega_{n}$. The first critical speeds in Y and X directions based on the parameters of Table 3 were calculated to be around 2197 rpm and 2307 rpm, respectively. The results indicate a nearly symmetric equivalent mass in both directions, whereas differences in stiffness and damping lead to distinct natural frequencies, with the Y direction exhibiting a lower natural frequency than the X direction. These identified parameters establish the physical foundation for interpreting the measured vibration responses. Figure 7a shows one case of the vibration signals by the laser displacement sensors obtained after applying a 10 Hz filter. In the present rotor system, the first natural frequency is well separated from the higher vibration modes. As a result, the measured response is predominantly governed by the 1X component generated by rotor imbalance and shaft-bow, both of which inherently excite synchronous (1X) vibration. Therefore, the signals in Figure 7a effectively represent the 1X response of the rotor system. The absolute phase information with respect to the KP is shown in Figure 7b, in which the abscissa unit is converted from time *t* to angular position *θ* (degrees) according to the relation θ = ωt/2π × 360°. As expected, the Y-phase is clearly observed to lag approximately 90° behind the X-phase.

After acquiring the displacement responses in the X and Y directions, these data are subsequently converted into four fault-related features $f_{4\times 1}={\left\{f_{1},f_{2},f_{3},f_{4}\right\}}^{T}$ according to the relationships defined in Equations (10)–(13). These four quantities constitute a 4-dimensional feature vector $f_{4\times 1}$, which is used as the input to FNN. The network outputs a 4-dimensional vector of fault components ${\left\{U_{x},U_{y},S_{x},S_{y}\right\}}^{T}$. These fault components are then transformed into the corresponding physical fault magnitudes and phase angles, namely the imbalance magnitude U and angle α, and the shaft-bow magnitude s and angle θ.

#### 2.3.2. Diagnosis Result of Experimental Data

Table 4 and Table 5 summarize the diagnostic results obtained for the experimental cases operated at 1500 and 2500 rpm, respectively. It can be observed that at the sub-critical speed of 1500 rpm relatively large discrepancies exist between the diagnosed and induced fault parameters for both amplitude- and phase-related quantities. For the imbalance amount U, the errors range from 26.68% to 38.91% across different phase configurations, while the shaft-bow amplitude s is consistently overestimated, with errors exceeding 23% in all cases. Moreover, significant angular deviations are observed for both the imbalance angle α and the shaft-bow angle θ, with phase errors reaching up to 31.88° and 24.85°, respectively.

As the rotor operates at the trans-critical speed of 2500 rpm, as shown in Table 5, the diagnosed imbalance magnitude U errors vary from about 5% to 28%, depending on the phase configuration. Meanwhile, the shaft-bow amplitude s is still systematically overestimated, with errors generally exceeding 17% in all cases. In addition, considerable angular deviations persist for both the imbalance α and shaft-bow θ, with phase errors reaching values above 20° for several configurations.

A similar behavior result was reported in [35]; the application of this methodology to experimental data revealed substantial inconsistencies between diagnosed and induced fault values. For example, in [35], at sub-critical speed (1600 rpm, shaft-bow *s* = 0.5 mm), the errors became more pronounced: imbalance estimation errors remained between 5.3% and 26.7%, but shaft-bow errors escalated to 38% in most configurations, accompanied by angular errors exceeding 40°. Under trans-critical speed of 3200 rpm, shaft-bow *s* = 0.5 mm, imbalance errors ranged from only 2.4% in the in-phase case to as high as 27.5% in the perpendicular case, while shaft-bow errors varied between 16.0% and 30.0%, with angular deviations reaching up to 15°. At severe shaft-bow of *s* = 4 mm at low speed (650 rpm), imbalance errors increased dramatically, reaching 23.8% in-phase and 26.0% in the anti-phase case, whereas shaft-bow errors remained comparatively smaller (only 4.2–7.0%). These large deviations-sometimes surpassing 30% in magnitude or 50° in angular phase-clearly demonstrate the limitations of the previous methodology and its vulnerability to speed-dependent and bow-dominated dynamics.

An explanation for these discrepancies is the presence of inherent faults, which already exist within the mechanical system but are neither explicitly identified nor accounted for during the construction of the physical model or the vibration data acquisition process. These inherent forces introduce systematic deviations between the real system and its idealized representation, thereby degrading the diagnostic accuracy of conventional hybrid approaches. The existence of such inherent faults, together with the observed limitations in the diagnostic results, provides the primary motivation for the present study. Accordingly, an improved hybrid framework with a self-compensation mechanism is proposed to explicitly address the influence of inherent faults, with the aim of reducing the errors observed in previous approaches and achieving more accurate diagnosis of simultaneous imbalance and shaft-bow.

## 3. Self-Compensation Algorithm

In this study, we propose a compensation-based identification approach aimed at determining the inherent faults of a multi-fault rotor system, specifically addressing the coexistence of imbalance and shaft-bow. By integrating analytical modeling techniques with machine learning, the proposed methodology not only enables the accurate estimation of intrinsic fault parameters but also enhances the reliability and robustness of the diagnostic process under complex operating conditions.

**Figure 8:**
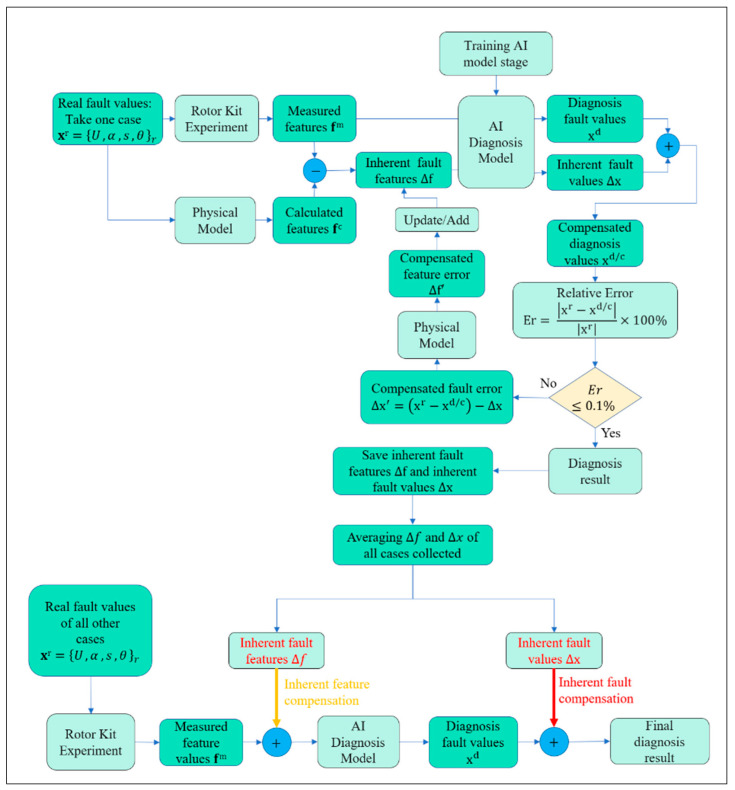
The flowchart of the self-compensation method.

After completing the AI model construction, the model is used to diagnose real faults in the self-compensation stage, as shown in Figure 8. This phase begins by selecting a representative fault case and applying it to the rotor test rig to obtain the measured vibration features, while the same fault parameters are also introduced into the physical rotor model to compute the corresponding calculated features. The difference between the measured and calculated features is then attributed to and extracted as the inherent fault, which reflects the unmodeled physical effects and structural uncertainties of the system. These features are subsequently fed into the AI diagnosis model to generate an inherent estimate of the fault parameters, after which a compensation mechanism is introduced to reduce prediction bias. The compensated result is iteratively evaluated against the induced fault values using the relative error criterion, and the compensation loop continues until the error falls below a specified threshold (e.g., 0.1% in our case). Once convergence is achieved, the inherent fault features and the corresponding inherent fault correction terms are stored for subsequent diagnoses. In the second stage, when a new fault case is introduced for diagnosis, the compensation knowledge accumulated during the first phase is systematically reused to enhance the accuracy of the prediction. Specifically, the inherent fault values and inherent feature sets identified during the iterative compensation process of the reference case are applied to the newly measured dataset to mitigate the influence of structural uncertainties, modeling errors, and unmodeled physical effects. Depending on the type of compensation utilized, two distinct diagnostic strategies are defined. When the measured features of the new case are compensated by adding the previously extracted inherent feature set, the procedure is referred to as the Inherent Feature-based Compensation (IFbC) algorithm. This approach assumes that the inherent discrepancies between the physical system and the mathematical model primarily manifest in the feature domain and can be systematically corrected by shifting the new feature vector according to the inherent feature error obtained earlier. In contrast, when compensation is performed by adjusting the predicted fault parameters using the pre-identified inherent fault values, the method is termed Inherent Fault Compensation (IFC). This strategy directly corrects the model output in the parameter domain, under the assumption that the systematic bias is more consistently represented as an offset in the estimated fault parameters rather than in the feature space.

A comprehensive comparison between IFC and IFbC—including their effectiveness in reducing diagnostic error, their robustness and their sensitivity to variations in fault magnitude—is presented in Section 4. Through this combined process, which integrates physical modeling, experimental measurement, AI-based regression, and iterative compensation, the proposed framework provides a robust and highly accurate solution for diagnosing rotor systems with coupled imbalance and shaft-bow.

## 4. Experimental Validation and Results Discussion

**Figure 9:**
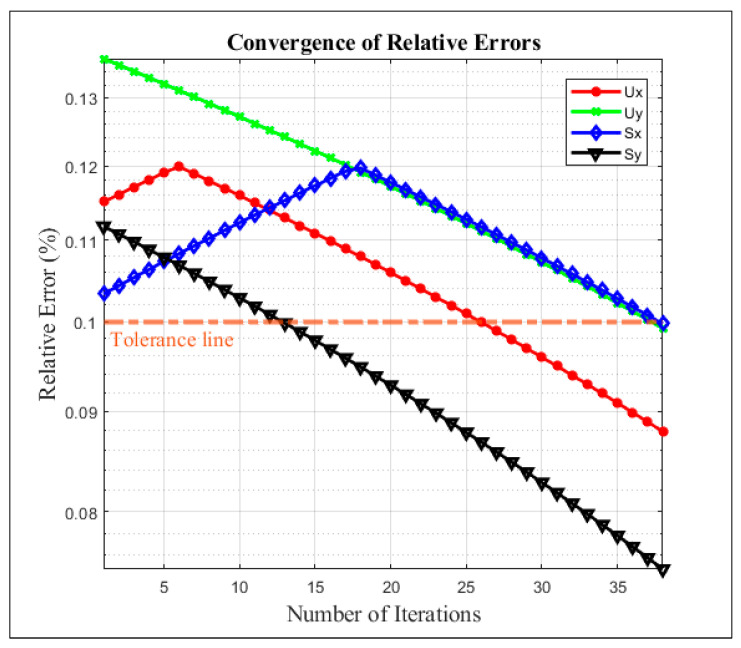
Convergence of relative errors for Same quadrant case operated at 1500 rpm.

In this section, the Same quadrant case was first selected to find the inherent fault parameters. Figure 9 illustrates the convergence behavior of the proposed physics–AI integrated diagnostic framework under a tolerance threshold of 0.1%. Due to the incorporation of the physical model, the relative errors at the first iteration are already constrained to a low level, ranging from 0.114% to 0.136%, indicating an effective physics-based initialization that significantly narrows the solution space of the AI diagnostic model. Although the gap between the maximum initial error and the tolerance value is relatively small (approximately 0.036%), a total of 38 iterations is required to ensure that all four diagnostic variables simultaneously satisfy the convergence criterion.

This gradual convergence is physically reasonable, as the proposed framework employs an incremental compensation strategy. At each iteration, only a small amount of fault feature—called compensated feature correction $∆f^{\prime }$—is added to the inherent fault features ∆f, thereby avoiding overcompensation and preserving the physical consistency of the diagnostic process. Moreover, the relative error curves exhibit an approximately linear decreasing trend, reflecting a stable and well-coordinated interaction between the physical model and the AI-based regression. These results demonstrate that the proposed hybrid approach achieves reliable convergence while maintaining both diagnostic accuracy and physical interpretability.

Table 6 presents the diagnostic results obtained using the proposed self-compensation algorithm. The estimated fault parameters show good agreement with the reference values for all test fault values. The errors of both imbalance and shaft-bow quantities and angular positions remain below the tolerance value, confirming the generalization capability of the proposed framework.

As shown in Table 7, the identified inherent deviations are mainly reflected in the angular components of the imbalance and shaft-bow faults, while the corresponding amplitude deviations remain small. This indicates that the dominant systematic discrepancies in the experimental rig are primarily phase-related rather than magnitude related. In addition, the inherent feature components are on the order of $10^{-4}$, suggesting that even small deviations in the feature domain can introduce noticeable bias in fault estimation if they are not explicitly compensated.

By applying the same iterative process to the other three cases, namely, in-phase, anti-phase, and perpendicular, the individual and averaged inherent fault parameters and inherent feature components across all cases are summarized in Table 8. This provides a representative description of the systematic discrepancies between the physical system and the mathematical model. The averaged inherent angles of imbalance and shaft-bow, approximately 116° and 98°, respectively, together with near-zero amplitude values further confirm the directional nature of the inherent faults. The averaged inherent features remain stable and on the same order of magnitude, demonstrating their suitability as prior compensation information.

Following the same procedure, the inherent fault parameters and inherent feature components for the cases operated at 2500 rpm are summarized in Table 9. Similar to the results obtained at 1500 rpm, the estimated inherent imbalance and shaft-bow amplitudes remain relatively small, while their corresponding angles exhibit consistent directional characteristics across the different phase configurations. The averaged inherent imbalance and shaft-bow angles are approximately 112° and 89°, respectively, indicating a stable orientation of the inherent faults within the rotor system.

Furthermore, when comparing the inherent fault values obtained at 1500 rpm and 2500 rpm, no significant differences are observed in either the magnitude or the directional characteristics of the estimated parameters. The inherent feature components also remain within the same order of magnitude across both operating conditions. This consistency indicates that the identified inherent faults are not random artifacts caused by a specific operating speed, but rather represent stable baseline characteristics of the rotor system. Such stability suggests that the proposed identification approach is robust with respect to changes in rotational speed. Therefore, the averaged inherent fault and feature values can be reliably used as prior compensation parameters to enhance diagnostic accuracy under different operating conditions.

Table 10 presents the compensated diagnostic results for the cases operated at 1500 rpm. After applying the compensation strategies, a clear improvement in diagnostic accuracy can be observed for all fault parameters. The IFbC method reduces the imbalance magnitude errors to about 7–18% and the shaft-bow amplitude errors to approximately 5–9%, while the angular deviations are significantly decreased, with most imbalance angle errors below 12° and bow orientation errors within about 1–5°. The IFC approach yields the most accurate results, further lowering the imbalance magnitude errors to around 2–14% and maintaining the shaft-bow amplitude errors near 5–6%. The angular parameters are identified with much higher precision, with imbalance angle errors as low as 0.14° and shaft-bow orientation errors generally within 0.5–1.8°.

A similar trend can be observed in Table 11 for the cases operated at 2500 rpm. After applying the compensation strategies, the diagnostic accuracy is significantly improved for all investigated fault parameters. In particular, the IFbC already reduces the errors of the imbalance magnitude to approximately 16–21%, while the phase errors decrease to only about 0.7–3°. A further improvement is achieved using the IFC scheme. With this method, the imbalance magnitude errors are reduced to approximately 1.33–6.75%, and the shaft-bow amplitude errors decrease to about 2–8%. More importantly, the angular deviations are substantially minimized, with the imbalance angle errors falling to around 1.24–2.89° and the shaft-bow orientation errors remaining within 0.24–2.43°.

Introducing compensation markedly improves performance, and the IFC scheme consistently outperforms IFbC in all cases. With IFC, the imbalance magnitude error is reduced to about 13–20%, the shaft-bow amplitude error drops to roughly 4.6–6.6%, and the angular errors decrease to approximately 0.45–2.20° for the imbalance angle and 0.14–2.20° for the bow orientation, representing a reduction by roughly an order of magnitude compared with the uncompensated case for the phase variables. These results demonstrate that IFC is more effective than IFbC in mitigating the influence of inherent faults and achieving accurate estimation of imbalance and shaft-bow parameters.

**Figure 10:**
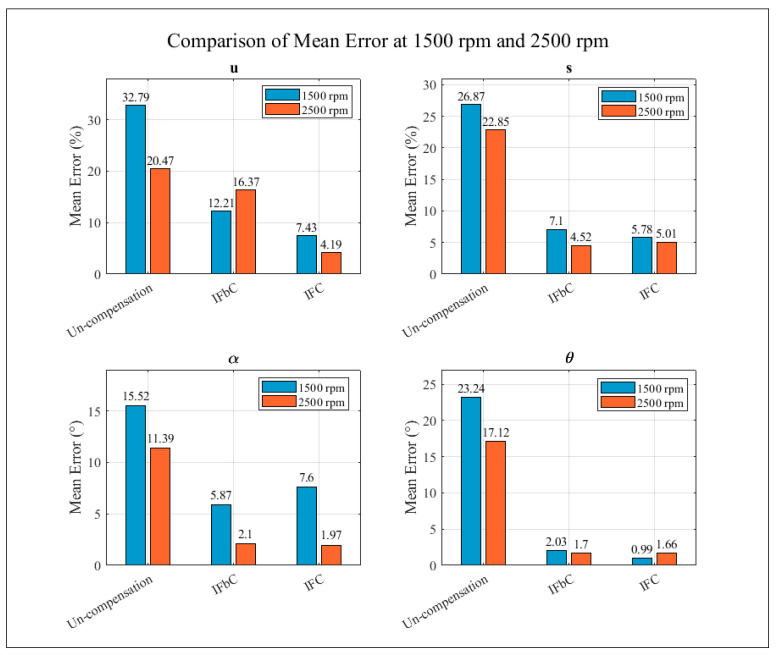
Mean error values for four fault components operated at 1500 rpm and 2500 rpm.

Figure 10 presents a comparison of the mean diagnostic errors for the four fault parameters at 1500 rpm and 2500 rpm under three different strategies: no-compensation, IFbC, and IFC. For all parameters, the no-compensation case exhibits the highest mean errors, indicating limited diagnostic accuracy when finding inherent faults or inherent features is not addressed. After applying the IFbC method, the mean errors are significantly reduced for both amplitude and phase variables.

The IFC strategy provides the best performance, yielding the lowest mean errors in most cases. For example, the mean error of the imbalance magnitude decreases from 32.79% to 7.43% at 1500 rpm and from 20.47% to 4.19% at 2500 rpm, while the shaft-bow amplitude error is reduced from 26.87% to 5.78% and 22.85% to 5.01%, respectively. Similar improvements are observed for the angular parameters, where the mean errors of the imbalance angle and bow orientation drop to below 8° and 1–2°, respectively. The consistent reduction of errors at both rotational speeds demonstrates that the proposed self-compensation framework effectively mitigates the influence of inherent faults and maintains robust diagnostic performance under different operating conditions.

It should be emphasized that the experimental validation in this study is performed under controlled laboratory conditions, where external loads, structural uncertainties, and environmental noise are not fully represented. As a result, the proposed method is validated within a simplified framework, and its performance in real industrial applications may be influenced by additional complexities. Future work will focus on extending the proposed approach to more realistic operating environments.

## 5. Conclusions

The present work proposed a hybrid diagnostic methodology that integrates a Jeffcott rotor model, a feed-forward neural network and a self-compensation algorithm to address the problem of imbalance and shaft-bow in rotor–bearing systems with inherent faults. By combining physics-based feature generation with data-driven regression and iterative compensation, the method enabled accurate estimation of four fault parameters using a limited number of experimental measurements, while maintaining physical interpretability of the diagnostic process. Experimental implementation on an RK4 Bently Nevada rig with controlled imbalance and shaft-bow conditions demonstrated stable convergence of the compensation loop and good agreement between the identified and reference fault values for all test cases.

A detailed analysis of the inherent faults and inherent features further revealed that the dominant systematic discrepancies between the mathematical model and the physical rotor rig are primarily phase-related. These discrepancies can be represented by a relatively stable inherent feature pattern that remains consistent across different operating conditions. Two compensation strategies were investigated, namely the Inherent Feature-based Compensation (IFbC) and the Inherent Fault Compensation (IFC) schemes, and both approaches significantly reduced the diagnostic errors compared with the uncompensated hybrid method. Among them, IFC achieved the lowest mean errors for all four fault parameters, particularly in reducing the angular deviations, indicating that compensation performed directly in the fault domain is highly effective in mitigating the influence of inherent faults and structural uncertainties.

Furthermore, the self-compensation algorithm was shown to improve diagnostic accuracy under both sub-critical and trans-critical operating speeds (1500 rpm and 2500 rpm). The consistent performance improvement across these different dynamic regimes confirms the robustness of the proposed method. These findings demonstrate that the self-compensated hybrid framework provides a reliable approach for practical fault diagnosis of rotating machinery. Therefore, the proposed methodology shows strong potential for integration into real-time condition monitoring and prognostic systems for industrial rotating machines. Future work will extend this approach to more complex rotor configurations, additional fault types, and time-varying operating conditions.

## Figures and Tables

**Table 1:** The average RMSE of four fault components at testing stage.

Fault Component	$U_{x}$	$U_{y}$	$S_{x}$	$S_{y}$
Average RMSE	$2.37\times 10^{-5}$	$7.22\times 10^{-5}$	$2.44\times 10^{-6}$	$3.07\times 10^{-6}$

**Table 2:** Fault parameters of 4 fault cases.

Case	Induced Fault Values			
Same quadrant	U	α	s	θ
	6	67.5°	0.5	45°
In-phase	U	α	s	θ
	6	45°	0.5	45°
Anti-phase	U	α	s	θ
	12	225°	0.5	45°
Perpendicular	U	α	s	θ
	12	135°	0.5	45°

**Table 3:** Estimated model parameters of the experimental rig.

	$M_{eq}(kg)$	ζ	$K_{s}(kN/m)$	$K_{b}(kN/m)$	$K_{eq}(kN/m)$	$\omega_{n}(rad/s)$
Y	0.96	0.47%	96.908	108.92	51.282	230.1
X	0.96	0.5%	96.908	135.72	56.538	241.6

**Table 4:** Diagnosis values for experimental cases operated at 1500 rpm.

Case	Induced Fault	Value	Diagnosed	Error
Same quadrant	U	6	8.19	36.48%
	α	67.5°	76.96°	9.46°
	s	0.5	0.64	28.79%
	θ	45°	69.06°	24.06°
In-phase	U	6	8.33	38.91%
	α	45°	55.99°	10.99°
	s	0.5	0.64	27.51%
	θ	45°	69.43°	24.43°
Anti-phase	U	12	15.20	26.68%
	α	225°	234.76°	9.76°
	s	0.5	0.64	27.23%
	θ	45°	69.85°	24.85°
Perpendicular	U	12	15.49	29.10%
	α	135°	166.88°	31.88°
	s	0.5	0.62	23.96%
	θ	45°	64.61°	19.61°

**Table 5:** Diagnosis values for experimental cases operated at 2500 rpm.

Case	Induced Fault	Value	Diagnosed	Error
Same quadrant	U	6	7.37	22.83%
	α	67.5°	72.86°	5.36°
	s	0.5	0.61	21.74%
	θ	45°	61.92°	16.92°
In-phase	U	6	7.68	28.04%
	α	45°	52.31°	7.31°
	s	0.5	0.64	27.58%
	θ	45°	63.47°	18.47°
Anti-phase	U	12	14.18	18.17%
	α	225°	232.63°	7.63°
	s	0.5	0.59	18.43%
	θ	45°	62.71°	17.71°
Perpendicular	U	12	13.54	12.83%
	α	135°	160.27°	25.27°
	s	0.5	0.62	23.64%
	θ	45°	60.38°	15.38°

**Table 6:** Diagnosis values for Same quadrant case operated at 1500 rpm.

Case	Induced Fault Value		Diagnosed	
	Fault	Value	Diagnosed	Error
Same quadrant	U	6.0	6.00	0.06%
	α	67.5°	67.49°	0.01°
	s	0.5	0.50	0.02%
	θ	45.0°	45.03°	0.03°

**Table 7:** Inherent faults and inherent features for the Same quadrant case operated at 1500 rpm after iterative process.

**Inherent Fault Values**			
ΔU	Δα	Δs	Δθ
0.018	97.29°	0.004	104.86°
**Inherent feature values**			
$f_{1}$	$f_{2}$	$f_{3}$	$f_{4}$
2.00 × 10^−4^	8.32 × 10^−4^	8.15 × 10^−4^	1.98 × 10^−4^

**Table 8:** Average values of inherent fault and inherent feature for cases operated at 1500 rpm.

Case	Inherent Fault Values				Inherent Feature Values			
	ΔU	Δα	Δs	Δθ	$f_{1}$	$f_{2}$	$f_{3}$	$f_{4}$
Same quadrant	0.018	97.29°	0.004	104.86°	2.00 × 10^−4^	8.32 × 10^−4^	8.15 × 10^−4^	1.98 × 10^−4^
In-phase	0.027	105.27°	0.002	127.34°	5.73 × 10^−4^	8.88 × 10^−4^	8.64 × 10^−4^	5.66 × 10^−4^
Anti-phase	0.013	121.12°	0.002	120.02°	1.30 × 10^−4^	7.96 × 10^−5^	8.74 × 10^−5^	1.26 × 10^−4^
Perpendicular	0.012	139.46°	0.001	38.73°	5.24 × 10^−5^	3.83 × 10^−5^	4.49 × 10^−5^	6.00 × 10^−5^
Average	0.018	115.79°	0.002	97.74°	2.39 × 10^−4^	4.59 × 10^−4^	4.53 × 10^−4^	2.38 × 10^−4^

**Table 9:** Average values of inherent fault and inherent feature for cases operated at 2500 rpm.

Case	Inherent Fault Values				Inherent Feature Values			
	ΔU	Δα	Δs	Δθ	$f_{1}$	$f_{2}$	$f_{3}$	$f_{4}$
Same quadrant	0.023	81.50°	0.008	95.64°	7.94 × 10^−4^	5.38 × 10^−4^	−5.19 × 10^−4^	7.91 × 10^−4^
In-phase	0.018	109.74°	0.003	111.48°	3.72 × 10^−4^	−1.81 × 10^−4^	1.67 × 10^−4^	3.64 × 10^−4^
Anti-phase	0.012	121.29°	0.002	119.01°	−1.44 × 10^−4^	−1.00 × 10^−5^	1.09 × 10^−5^	−1.39 × 10^−4^
Perpendicular	0.012	134.48°	0.000	29.89°	−3.05 × 10^−4^	−6.58 × 10^−4^	5.83 × 10^−5^	−2.32 × 10^−5^
Average	0.016	111.75°	0.003	89.01°	1.79 × 10^−4^	−7.78 × 10^−5^	−7.07 × 10^−5^	2.48 × 10^−4^

**Table 10:** Final diagnosis values for cases operated at 1500 rpm.

Case	Induced Fault Values		No-Compensation		IFbC		IFC	
	Fault	Value	Diagnosed	Error	Diagnosed	Error	Diagnosed	Error
Same quadrant	U	6	8.19	36.48%	6.83	13.78%	6.79	13.14%
	α	67.5°	76.96°	9.46°	72.28°	4.78°	78.80°	11.30°
	s	0.5	0.64	28.79%	0.45	9.25%	0.47	5.86%
	θ	45°	69.06°	24.06°	43.68°	1.32°	44.55°	0.45°
In-phase	U	6	8.33	38.91%	6.46	7.68%	6.74	12.41%
	α	45°	55.99°	10.99°	56.83°	11.83°	59.28°	14.28°
	s	0.5	0.64	27.51%	0.46	8.14%	0.47	5.63%
	θ	45°	69.43°	24.43°	43.98°	1.02°	45.51°	0.51°
Anti-phase	U	12	15.20	26.68%	9.80	18.37%	12.24	1.97%
	α	225°	234.76°	9.76°	218.38°	6.62°	224.86°	0.14°
	s	0.5	0.64	27.23%	0.47	5.11%	0.47	5.57%
	θ	45°	69.85°	24.85°	40.35°	4.65°	46.18°	1.18°
Perpendicular	U	12	15.49	29.10%	10.92	9.02%	12.26	2.20%
	α	135°	166.88°	31.88°	134.77°	0.23°	130.32°	4.68°
	s	0.5	0.62	23.96%	0.53	5.90%	0.47	6.05%
	θ	45°	64.61°	19.61°	46.14°	1.14°	43.19°	1.81°

**Table 11:** Final diagnosis values for cases operated at 2500 rpm.

Case	Induced Fault Values		No-Compensation		IFbC		IFC	
	Fault	Value	Diagnosed	Error	Diagnosed	Error	Diagnosed	Error
Same quadrant	U	6	7.37	22.83%	5.56	7.33%	6.21	3.50%
	α	67.5°	72.86°	5.36°	70.69°	3.19°	69.48°	1.98°
	s	0.5	0.61	21.74%	0.47	6.01%	0.48	4.03%
	θ	45°	61.92°	16.92°	44.26°	0.74°	47.43°	2.43°
In-phase	U	6	7.68	28.04%	4.75	20.83%	6.31	5.16%
	α	45°	52.31°	7.31°	47.34°	2.23°	42.11°	2.89°
	s	0.5	0.64	27.58%	0.48	4.02%	0.51	2.00%
	θ	45°	63.47°	18.47°	46.11°	1.11°	45.24°	0.24°
Anti-phase	U	12	14.18	18.17%	10.01	16.58%	11.19	6.75%
	α	225°	232.63°	7.63°	224.17°	0.83°	226.78°	1.78°
	s	0.5	0.59	18.43%	0.47	6.02%	0.47	6.01%
	θ	45°	62.71°	17.71°	42.11°	2.89°	43.31°	1.69°
Perpendicular	U	12	13.54	12.83%	9.51	20.75%	11.84	1.33%
	α	135°	160.27°	25.27°	137.16°	2.16°	136.24°	1.24°
	s	0.5	0.62	23.64%	0.49	2.03%	0.46	8.00%
	θ	45°	60.38°	15.38°	47.04°	2.04°	42.72°	2.28°

## Data Availability

The data presented in this study is available on request from the corresponding author due to privacy.

## Nomenclature

The following symbols are used in this manuscript:

u(t),v(t)	Displacement of the disk center in the horizontal (X) and vertical (Y) directions (m)
M	Mass of the disk (kg)
m	Imbalance mass attached to the disk (kg)
$M_{T}$	The total mass (kg)
$M_{eq}$	Equivalent mass of rotor rig (kg), to replace *M*
e	Eccentricity distance between imbalance mass and disk center (m)
U	Imbalance magnitude (g.mm), *U = me*
$K_{x},K_{y}$	Equivalent lateral stiffness of shaft-bearings in X and Y direction (kN/m)
$K_{eq}$	Equivalent stiffness of the rotor rig (kN/m), to replace $K_{x},K_{y}$
$B_{x},B_{y}$	Equivalent lateral damping coefficients in X and Y directions (Ns/m)
s	Inherent shaft-bow amplitude (m)
α	Imbalance angular position with respect to key phasor (°)
θ	Shaft-bow angular orientation with respect to key phasor (°)
Ω	Rotor angular speed (rad/s)
$\omega_{nx},\omega_{ny}$	Natural frequency in X and Y directions (rad/s)
$\zeta_{x},\zeta_{y}$	Damping ratio in X and Y directions
$\tau_{x},\tau_{y}$	Frequency ratio in X and Y directions
$\lambda_{x},\lambda_{y}$	Phase lag in X and Y directions (rad)
$A_{x},A_{y}$	Amplitude amplification factors in X and Y directions
$f_{1},f_{2},f_{3},f_{4}$	Response features
$f_{4\times 1}$	Feature vector
$m_{4\times 1}$	Fault parameter vector
$U_{x},U_{y}$	Imbalance fault component in X and Y directions
$S_{x},S_{y}$	Shaft-bow fault component in X and Y directions
